# Time-Limited Trials Among Critically Ill Patients With Advanced Medical Illnesses to Reduce Nonbeneficial Intensive Care Unit Treatments: Protocol for a Multicenter Quality Improvement Study

**DOI:** 10.2196/16301

**Published:** 2019-11-25

**Authors:** Dong Chang, Jennifer Parrish, Nader Kamangar, Janice Liebler, May Lee, Thanh Neville

**Affiliations:** 1 Los Angeles BioMedical Research Institute Harbor-University of California Los Angeles Medical Center Torrance, CA United States; 2 Olive View Medical Center Sylmar, CA United States; 3 Los Angeles County-University of Southern California Medical Center Los Angeles, CA United States; 4 Division of Pulmonary and Critical Care Medicine University of California Los Angeles School of Medicine Los Angeles, CA United States

**Keywords:** intensive care, critical care, prognosis, outcome, prediction, medical uncertainty, medical futility, palliative care, communication

## Abstract

**Background:**

Invasive intensive care unit (ICU) treatments for patients with advanced medical illnesses and poor prognoses may prolong suffering with minimal benefit. Unfortunately, the quality of care planning and communication between clinicians and critically ill patients and their families in these situations are highly variable, frequently leading to overutilization of invasive ICU treatments. Time-limited trials (TLTs) are agreements between the clinicians and the patients and decision makers to use certain medical therapies over defined periods of time and to evaluate whether patients improve or worsen according to predetermined clinical parameters. For patients with advanced medical illnesses receiving aggressive ICU treatments, TLTs can promote effective dialogue, develop consensus in decision making, and set rational boundaries to treatments based on patients’ goals of care.

**Objective:**

The aim of this study will be to examine whether a multicomponent quality-improvement strategy that uses protocoled TLTs as the default ICU care-planning approach for critically ill patients with advanced medical illnesses will decrease duration and intensity of nonbeneficial ICU care without changing hospital mortality.

**Methods:**

This study will be conducted in medical ICUs of three public teaching hospitals in Los Angeles County. In Aim 1, we will conduct focus groups and semistructured interviews with key stakeholders to identify facilitators and barriers to implementing TLTs among ICU patients with advanced medical illnesses. In Aim 2, we will train clinicians to use protocol-enhanced TLTs as the default communication and care-planning approach in patients with advanced medical illnesses who receive invasive ICU treatments. Eligible patients will be those who the treating ICU physicians consider to be at high risk for nonbeneficial treatments according to guidelines from the Society of Critical Care Medicine. ICU physicians will be trained to use the TLT protocol through a curriculum of didactic lectures, case discussions, and simulations utilizing actors as family members in role-playing scenarios. Family meetings will be scheduled by trained care managers. The improvement strategy will be implemented sequentially in the three participating hospitals, and outcomes will be evaluated using a before-and-after study design. Key process outcomes will include frequency, timing, and content of family meetings. The primary clinical outcome will be ICU length of stay. Secondary outcomes will include hospital length of stay, days receiving life-sustaining treatments (eg, mechanical ventilation, vasopressors, and renal replacement therapy), number of attempts at cardiopulmonary resuscitation, frequency of invasive ICU procedures, and disposition from hospitalization.

**Results:**

The study began in August 2017. The implementation of interventions and data collection were completed at two of the three hospitals. As of September 2019, the study was at the postintervention stage at the third hospital. We have completed focus groups with physicians at each medical center (N=29) and interviews of family members and surrogate decision makers (N=18). The study is expected to be completed in the first quarter of 2020, and results are expected to be available in mid-2020.

**Conclusions:**

The successful completion of the aims in this proposal may identify a systematic approach to improve communication and shared decision making and to reduce nonbeneficial invasive treatments for ICU patients with advanced medical illnesses.

**International Registered Report Identifier (IRRID):**

DERR1-10.2196/16301

## Introduction

### Background

#### Clinical Significance of Intensive Care Unit Overutilization Among Patients With Advanced Medical Illnesses

In the United States, 1 in 5 people die using intensive care unit (ICU) services, frequently receiving invasive treatments despite minimal anticipated benefit [1,2]. Investigators in our research group found that 20% of ICU patients in the University of California Los Angeles (UCLA) health care system were perceived by physicians to be receiving futile care [3]. A recent study from our research group [4] and previous work from others [5-7] also showed that hospitals that utilize ICUs more frequently were more likely to perform invasive procedures and have higher costs with no improvement in hospital mortality. Interestingly, most patients with advanced medical illnesses prefer not to receive such aggressive care at the end of life [1,8-12] but ICU care in this population is increasing [2,13]. This trend represents an important health care problem; a multicenter controlled study estimated that patients with advanced medical illnesses who died in ICUs spent an average of 8 days in undesirable states, such as being comatose or receiving mechanical ventilation [14]. Among conscious ICU patients who died, 50% experienced significant pain for more than half the time during the final week of life [14]. Furthermore, terminal hospitalizations account for 7.5% of total inpatient costs in the United States, with ICU care accounting for nearly 80% of these costs [1,15]. Overall, these findings show that optimizing ICU utilization among patients with advanced medical illnesses is an opportunity to improve the quality and efficiency of care in this high-risk, high-cost population.

#### Intensive Care Unit Overutilization in the Los Angeles County Department of Health Services

Like many health care systems, ICU overutilization is highly prevalent in the hospitals of the Los Angeles County (LAC) Department of Health Services (DHS), the second-largest public health care system in the United States. A recent study from our group showed that among 808 medical ICU patients at Harbor-UCLA Medical Center (HUMC), over 20% had reduced likelihoods of benefit from ICU care due to advanced medical illnesses, such as advanced dementia and metastatic cancer (see Table 1) [16]. Of these patients, 86% died or were discharged in severely compromised health states and only 6% were discharged home. This subset of patients is at the highest risk to have invasive treatments that prolong suffering without improving outcomes. As such, we focus this proposal on improving ICU utilization on these critically ill patients with advanced medical illnesses.

In order to understand why patients with advanced medical illnesses received ICU care so frequently, we conducted semistructured interviews of ICU physicians and nurses at LAC DHS hospitals. Key themes that emerged from these interviews were that health care providers do not comprehensively discuss prognoses, risks and benefits, and patient preferences for ICU treatments, which lead to inaccurate expectations from ICU care and unrealistic fears of prematurely forgoing potentially beneficial treatments. Barriers to effective shared decision making included (1) underappreciation of the value of ICU care planning, (2) lack of institutional standards and tools for ICU care planning, and (3) difficulty in scheduling meetings between providers and families.

**Table 1:** Priority levels of medical intensive care unit (ICU) admissions at Harbor-University of California Los Angeles Medical Center.

Priority	Description	Percentage, %
1	Critically ill, needing intensive treatment and monitoring that cannot be provided outside of ICUs	46.9
2	Not critically ill, but requiring close monitoring and potentially immediate intervention	23.4
3	Critically ill, but reduced likelihood of recovery because of underlying diseases or severity of acute illness	20.9
4	Not appropriate for ICU; equivalent outcomes achievable with non-ICU care	8.8

#### Facilitating Shared Decision Making Using Time-Limited Trials

Prior work has shown that developing ICU interventions that change physician behaviors, facilitate communication between providers and families, and improve patient care is challenging [14,17,18]. A large multicenter clinical trial—the Study to Understand Prognoses and Preferences for Outcomes and Risks of Treatment (SUPPORT)—did not improve the quality of end-of-life care by providing physicians with patients’ prognostic information and nurse-facilitated advanced-care planning [14]. A hypothesis for why SUPPORT failed to change outcomes is that it did not use a multifaceted, systems approach to changing physicians’ behaviors [19,20]. Furthermore, even when prognoses are understood and risks and benefits of ICU care are discussed, it is likely that clinicians, patients, and families frequently remain uncertain about the appropriateness of ICU care [21-23]. In such situations, the default decision in most ICUs is to pursue aggressive ICU treatments, often without reassessment of that decision [24].

For patients with advanced illnesses, this approach places them at risk for prolonged suffering with minimal anticipated benefit [3,4]. Time-limited trials (TLTs) are agreements between the clinicians and the patients and decision makers to use certain medical therapies over a defined period of time and evaluate whether patients improve or worsen according to predetermined clinical parameters [24]. TLTs involve detailed discussions of patients’ preferences for care, prognosis, and what would constitute clinical improvement based on patients’ values and preferences. Follow-up meetings are held to see if patients improve or worsen according to predetermined clinical parameters and next steps in care are negotiated based on these results [24]. TLTs promote regular structured dialogue between providers and patients and their families, promote consensus in decision making through iterative assessments of clinical trajectory, and set rational boundaries to treatments based on patients’ goals of care. TLTs have been used effectively for outpatient advanced-care planning in patients with end-stage kidney disease, stroke, cancer, and chronic obstructive pulmonary disease [25-30]. However, to our knowledge there have been no studies that examined their effectiveness in ICU patients. Schenker et al examined audio-recordings of ICU family meetings for 72 patients at high risk for death or severe impairment and found that TLTs were offered only 13% of the time [31]. When TLTs were offered, clinicians frequently did not discuss key elements, such as outcomes used to determine improvement, worsening, or possible next steps after the trial [31].

In summary, nonbeneficial treatments are frequently delivered in medical ICUs to patients with advanced medical illnesses. Although the appropriateness of ICU care in this population is subject to varying opinions, there is general consensus that intensity of ICU treatments should align with patients’ prognoses, preferences, and values [32]. Previous studies suggest that most patients with advanced medical illnesses, when informed of their therapeutic options, would forgo invasive treatments and would prefer palliative approaches [1,8-12]. Unfortunately, structured care planning between clinicians and critically ill patients and their families is infrequent [33,34]. TLTs are a promising, but underutilized, care-planning approach for ICU patients with advanced medical illnesses. An intervention utilizing TLTs as a default care-planning approach for ICU patients with advanced medical illnesses has the potential to address key barriers to shared decision making identified in our preliminary studies [35]. However, given the paucity of studies on their use in critically ill patients and the complexity of ICU communication, input from key stakeholders on how best to implement TLTs in this group is crucial.

### Objectives

#### Overview

**Figure 1:**
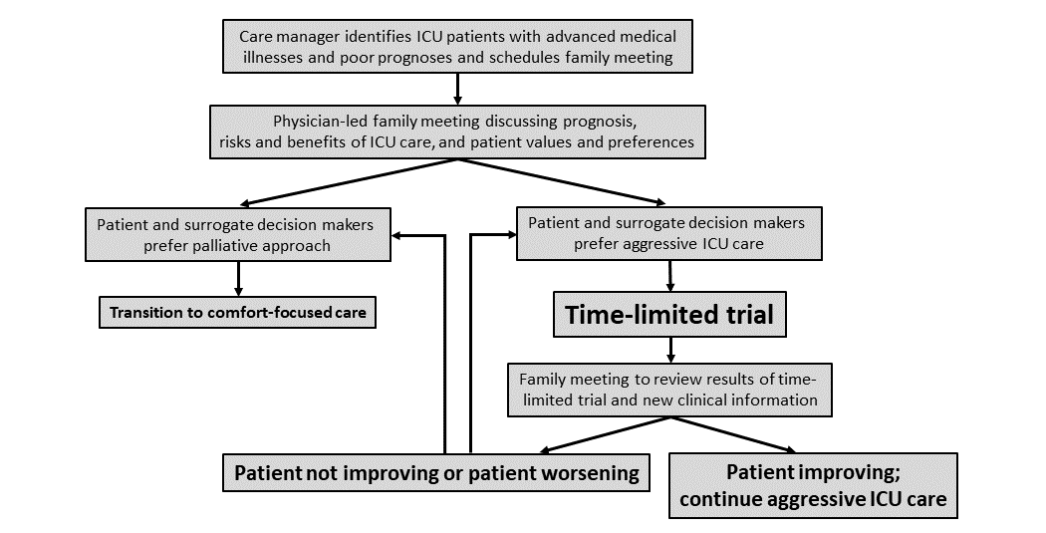
Implementation strategy for time-limited trials. ICU: intensive care unit.

The objective of this proposal is to test an intervention that seeks to reduce invasive and nonbeneficial ICU treatments by improving communication between providers, surrogate decision makers, and critically ill patients with advanced medical illnesses. We propose to implement an intervention that facilitates communication and shared decision making between providers and patients and their families by using protocoled TLTs for ICU patients with advanced medical illnesses who receive aggressive care (see Figure 1).

We hypothesize that a multicomponent quality-improvement intervention, informed by stakeholder input and that uses protocoled TLTs as the default ICU care-planning approach for critically ill patients with advanced medical illnesses, will decrease the duration and intensity of nonbeneficial ICU care without changing hospital mortality. We will examine our hypothesis with the specific aims discussed in the following sections.

#### Aim 1

Aim 1 consists of identifying barriers and facilitators to performing TLTs in ICU patients with advanced medical illnesses using focus groups of physicians and semistructured interviews of patients and their families.

#### Aim 2

Aim 2 consists of examining whether a multicomponent quality-improvement intervention using TLTs as the default care-planning approach for ICU patients with advanced medical illnesses reduces duration and intensity of nonbeneficial ICU treatments

## Methods

### Overview

This proposal will be conducted in the medical ICUs of three public hospitals in LAC DHS: HUMC, Olive View Medical Center (OVMC), and Los Angeles County-University of Southern California (LAC-USC) Medical Center. We will implement the aims sequentially across the medical centers. The sequential implementation strategy will be used to identify ways to improve training and uptake of the interventions with each iteration. For Aim 1, we will conduct focus groups and interviews with key stakeholders to identify facilitators and barriers to implementing TLTs among ICU patients with advanced medical illnesses. This will be performed prior to implementing quality-improvement interventions in Aim 2 (see Multimedia Appendix 1). The information obtained from these qualitative evaluations will be used to enhance our implementation strategy for Aim 2, in which we will examine the effectiveness of our intervention using a before-and-after study design (see Multimedia Appendix 1).

### Aim 1

#### Rationale and Overview

In the first phase of the proposed study, we will conduct focus groups with ICU physicians and interviews with patients and/or surrogate decision makers to explore attitudes toward ICU care among patients with advanced medical illnesses and strategies to optimally implement TLTs. We anticipate that themes from these sessions will include perceived barriers to conducting family meetings, knowledge and skill deficits among clinicians in conducting meetings, clinical outcomes most effective in decision making, optimal timing and frequency of meetings, optimal duration of TLTs, and communicating sensitive medical topics to lay populations.

#### Experimental Approach

##### Structure of Focus Groups With Intensive Care Unit Physicians

In-person focus groups with medical ICU physicians will be conducted at HUMC, OVMC, and LAC-USC Medical Center. We will invite all ICU attendings and fellows from each institution; we anticipate that 10-30 people will be invited and that 6-15 will participate per institution. Informed consent will be obtained for audio-recording. The meetings will be held at each medical center, will last approximately 90 minutes, and will be led by two of the study investigators.

##### Content of Focus Groups With Intensive Care Unit Physicians

Moderators will lead discussions in each group. Discussions will begin with an explanation of the risks and benefits of aggressive ICU care for patients with advanced medical illnesses and of the goal of delivering care that aligns with patients’ values and preferences. We will explain the concept of TLTs and elicit responses to a prespecified set of open-ended questions.

Sample questions include the following: (1) How are decisions regarding ICU care currently made? (2) Do you believe that TLTs are appropriate interventions? Why or why not? (3) What information is needed to effectively make decisions about continuing aggressive ICU care? (4) What is the best way to communicate that information? (5) Should care providers make recommendations regarding the next steps in care after TLTs? (6) What are barriers to having meetings with patients and their families? (7) What information do you typically provide in family meetings? (8) How do you feel about using a protocol and checklist during family meetings? (9) How comfortable are you with making recommendations for end-of-life care? and (10) How much variability do you perceive between physicians regarding prognoses and goals of care?

##### Structure of Semistructured Interviews With Patients and Surrogate Decision Makers

In-person interviews with patients and/or surrogate decision makers will be conducted at HUMC, OVMC, and LAC-USC Medical Center. We will invite English-speaking patients or family members and surrogate decision makers who are available to participate in interviews; we anticipate that that 20 people will be invited and that 10 will participate per institution. Patients and family members will be invited for interviews after at least 72 hours of ICU hospitalization to provide adequate opportunities for communication and care planning with ICU clinicians. Informed consent will be obtained for audio-recording. The interviews will be held at each medical center, will last approximately 45 minutes, and will be led by one of the study investigators.

##### Content of Semistructured Interviews With Patients and Surrogate Decision Makers

An investigator will lead semistructured interviews of patients and/or family members, which will explore decision making in the ICU. We will explore factors that played key roles in decision making during ICU hospitalization with a prespecified set of open-ended questions.

Sample questions will include the following: (1) Describe the most difficult decision you had to make during this hospitalization, (2) What decision did you make and how did you come to that decision? (3) What did the ICU care providers do that made that decision harder or easier? (4) What clinical events resonated with you regarding whether you or your family member was improving or worsening? (5) What other factors besides those discussed in family meetings affect your decision regarding ICU care? (6) How often were you confused about information that was presented to you during hospitalization? (7) What information was difficult to understand and why? and (8) What can the care providers do to better support you during this time?

#### Analysis of Focus Groups and Interviews

Analysis of data from focus groups and interviews will be descriptive, summarizing the range of issues that ICU physicians, patients, and surrogate decision makers discuss. Using the audio-recordings and moderators’ notes, content analysis of the group discussions and interviews will be performed to systematically define themes, emphasizing those that represent facilitators and barriers to implementation of our intervention [36-39]. These themes will be used to modify the implementation of the quality-improvement intervention in Aim 2.

#### Aim 2

##### Rationale and Overview

**Figure 2:**
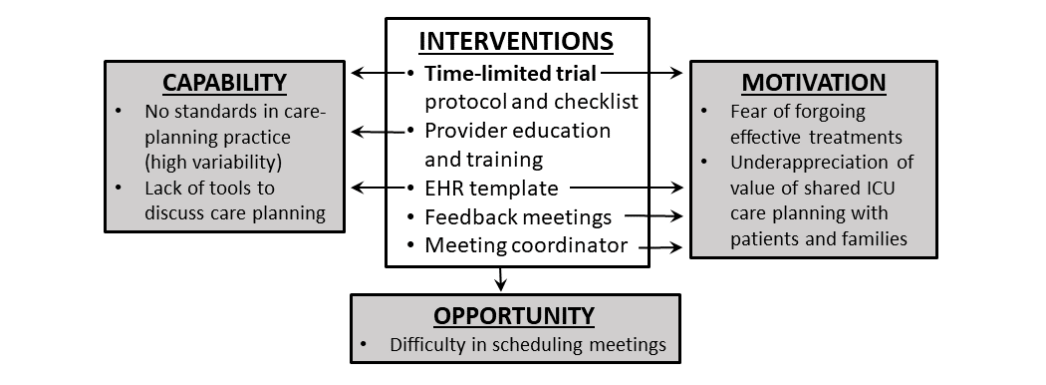
Conceptual framework for interventions. EHR: electronic health record; ICU: intensive care unit.

In the second phase of the proposed study, we will implement an intervention that facilitates family meetings using serial TLTs as the default care-planning approach for ICU patients with advanced medical illnesses. The multicomponent intervention is based on the *capability, opportunity, motivation to perform a behavior* (COM-B) framework by Michie and colleagues [40-42]; the intervention will address barriers identified in our preliminary studies that inhibit capabilities, opportunities, and motivation for effective shared decision making (see Figure 2).

##### Experimental Approach

###### Study Population

Patients with a low likelihood of benefitting from ICU care due to advanced medical illnesses will be identified by assigning patients to priority levels from the Society of Critical Care Medicine guidelines for ICU admissions (see Table 1) [43-45]. This system prioritizes ICU admissions based on the projected likelihood of benefit. For this proposal, we will train all ICU physicians—attendings and fellows—to perform daily priority-level assessments for ICU patients. We have previously published our experience with training ICU teams to classify patients using this system [16]. Each day, case managers will ask ICU physicians to assign priority levels to each patient after ICU rounds. Our intervention will be implemented on all new ICU admissions who are assigned to priority levels 3 or 4. We will exclude patients who were assigned to different priority levels on admission but who are assigned to priority levels 3 or 4 during their ICU stay. If patients cannot communicate for themselves and do not have surrogate decision makers, they will be excluded.

###### Setting and Study Design

The study will be conducted in medical ICUs of three LAC DHS hospitals using a before-and-after study design. The study will be conducted sequentially among the three hospitals to allow investigators to modify and improve the implementation strategy over the course of the study based on clinician feedback (see Multimedia Appendix 1). For each hospital, we will collect preintervention data during the first 4 months and examine outcomes for a 4-month period after implementing the study intervention (see Multimedia Appendix 1). There are 52 medical ICU beds among the three hospitals. We anticipate that 960 patients will be admitted and screened in both the pre- and postintervention periods across the three medical ICUs. Based on our preliminary data, we estimate that 15% will be priority 3 and 4 patients who are candidates for intervention and 10% will be excluded due to lack of surrogate decision makers. As such, we estimate studying 130 patients during each 4-month period. Using alpha=.05 (two-sided), beta=.20 (80% power), and mean ICU stay of 6.5 days (SD 3.7) from our previous study [16], we expect to be able to detect a difference of 1.3 ICU days between time periods.

###### Time-Limited Trial Protocol

Physician-led family meetings will be conducted using a standardized protocol, which includes: (1) introductions, (2) summary of ICU course, (3) discussion of short- and long-term prognosis, (4) risks and benefits of aggressive ICU care, and (5) eliciting patients’ preferences for care during critical illness and/or at the end of life (see Multimedia Appendix 2 and Figure 1). Based on discussion of these elements, if patients and surrogate decision makers wish for a palliative approach, the patient will be transitioned to comfort-focused care. If patients and surrogate decision makers prefer aggressive ICU care, a TLT will be performed. For the TLT, care providers and surrogate decision makers will identify specific clinical parameters that will be used to determine whether patients are improving or worsening. Care providers will recommend a time period for which these parameters will be followed and likely actions to be taken at the end of the trial based on improvement or worsening. In the follow-up meeting, care providers will review trends in clinical parameters; they will also redefine prognoses based on these trends and additional clinical information obtained since the last meeting. Based on this information, recommendations regarding the next steps for care will be made. If the patients and surrogate decision makers opt for a palliative approach, comfort-focused care will be provided. If they prefer continuation of aggressive care, another TLT will be negotiated. This iterative process will be continued, with sequential meetings performed at the discretion of the ICU team (see Figure 1).

###### Multicomponent Implementation Strategy

Additional components to the implementation strategy are shown in Figures 1 and 2. ICU physicians—attendings and fellows—will be trained to use the TLT protocol in educational sessions utilizing actors as family members in role-playing scenarios (see Figure 2). Case managers will identify eligible patients each day by asking ICU physicians which admissions are ranked priority 3 or 4 and will schedule family meetings between care providers and patients and their families within 24 hours of admission. Family meetings will be conducted using checklists to improve compliance with the protocol (see Multimedia Appendix 2). A standardized electronic health record (EHR) template will be created to encourage documentation of important outcomes from meetings and to facilitate data collection. Each medical ICU will have a physician champion who will direct implementation of study protocols at each medical center and conduct monthly feedback sessions with ICU teams to discuss opportunities for improvement. Physician champions and research team members will meet every month to discuss strategies to improve family meetings, patient enrollment, and data collection.

###### Outcomes and Measurements

All outcomes will be collected in both the pre- and postintervention periods. The primary outcome will be ICU length of stay. Secondary outcomes will include hospital length of stay, days receiving life-sustaining treatments (eg, mechanical ventilation, use of vasopressor medications, and renal replacement therapy), number of attempts at cardiopulmonary resuscitation (CPR), number of invasive procedures (eg, central venous or arterial catheterization, thoracentesis, paracentesis, lumbar puncture, and endoscopy), and outcomes of hospitalization (eg, death, discharge to hospice, skilled nursing facility, or home). These data will be collected from retrospective chart review by trained case managers using standardized data abstraction forms.

###### Analysis Plan

Primary and secondary outcomes will be compared before and after the intervention [46-48]. Days in ICU, days in hospital, and days receiving life-sustaining treatments will be compared using Wilcoxon rank-sum tests [49,50]. ICU and hospital mortality rates will be compared using χ^2^ tests. Other continuous and categorical outcomes will be compared between groups using *t* tests or χ^2^ tests, respectively, or equivalent nonparametric approaches for variables that are nonnormally distributed. Multivariable regression models will be used to examine the effects of covariates, such as patient demographics, comorbid conditions, severity of illness, and hospitals, on study outcomes [49,50]. Primary and secondary outcomes before and after the intervention will also be analyzed and will be stratified by ICU survivors and nonsurvivors. Days receiving ICU care, days receiving life-sustaining treatments, number of invasive procedures, and number of CPR attempts among patients who did not survive hospitalization will be considered nonbeneficial treatment and will be compared before and after the intervention.

###### Expected Results

We expect to find fewer ICU days, hospital days, and days of life-sustaining treatments after the TLT intervention. We also expect a fewer number of attempts at CPR and other invasive procedures. We expect mortality rates to remain unchanged before and after the intervention. We expect reductions in ICU days, days receiving life-sustaining treatments, and number of invasive procedures after the intervention to be greater among patients who died during hospitalization than among survivors.

###### Limitations

We recognize that priority levels used to identify patients are subjective. However, we believe that using clinicians’ general impressions on the likelihood of benefit from ICU care, rather than more objective measures such as prognostic scoring systems or predefined lists of medical conditions, emulates clinical practice and will be more informative regarding the effectiveness of this intervention. If there are fewer priority 3 and 4 patients than anticipated, we will expand the study criteria to include patients who were initially categorized into a different priority level on ICU admission but were assigned priority level 3 or 4 during the ICU hospitalization. Based on our preliminary data, this will make an additional 10% of ICU patients eligible for the study.

### Ethical Considerations

Our project was approved by the Institutional Review Board (IRB) at the Los Angeles BioMedical Research Institute at HUMC (project number: 043544) with approval at the other two medical centers using a reliance agreement. For Aim 1, informed consent was obtained for participants in focus groups and interviews. For Aim 2, the IRB waived the need for informed consent. There were several key factors involved in the waiver of consent. First, communication between ICU physicians and families is an expected practice; as well, the aim of the quality-improvement intervention was to encourage physicians to discuss elements that are vital to effective shared decision making, such as patient values and preferences, prognosis, and expectations for ICU treatments. As such, the intervention posed minimal risk to participants beyond usual ICU practice. Second, TLTs were not coercive or prescriptive, and maintained patient and family autonomy in decision making: at the end of the TLTs, patients and family members could choose to continue invasive treatments. Third, the quality-improvement intervention created a new default communication approach that applied to all ICU patients with advanced medical illnesses, regardless of participation in the study. Finally, implementation of the quality-improvement program was approved by the LAC DHS. Given these factors, the IRB determined that there was minimal risk to participants, waiver of consent would not adversely affect the rights and welfare of the participants, and the project could not be feasibly performed without the waiver.

## Results

The study began in August 2017. The implementation of interventions and data collection were completed at HUMC and OVMC. As of September 2019, the study was at the postintervention stage at the LAC-USC Medical Center. We have completed focus groups with physicians at each medical center (N=29) and interviews of family members and surrogate decision makers (N=18). The study is expected to be completed in the first quarter of 2020, and results are expected to be available in mid-2020.

## Discussion

Overutilization of ICU treatments among critically ill patients with advanced medical illnesses leads to medical care that is invasive, costly, and potentially misaligned with patient preferences. The successful completion of the aims in this proposal will improve the quality and efficiency of care by reducing unnecessary invasive treatments and decreasing ICU care that does not achieve its intended goals and prolongs suffering. This will be achieved through better communication and alignment of ICU care with patients’ values and preferences. Additionally, these studies will generate preliminary data and a track record of collaboration between researchers, clinicians, and hospital leaders, which will be foundational for future applications that attempt large-scale implementation of interventions that improve ICU communication and care planning. Thus, this proposal has the potential to catalyze the development of an ICU research program that addresses complex challenges in health care systems; this could be done through partnerships between academicians in health services research and frontline physicians and hospital administrators with experience in operationalizing health care improvements.

## Appendix

Multimedia Appendix 1 Study timeline.

Multimedia Appendix 2 Time-limited trial (TLT) family meeting protocol and checklist.

Multimedia Appendix 3 Peer-reviewer report from the UCLA Clinical and Translational Science Institute.

## Abbreviations

COM-B: capability, opportunity, motivation to perform a behavior
CPR: cardiopulmonary resuscitation
CTSI: Clinical and Translational Research Institute
DHS: Department of Health Services
EHR: electronic health record
HUMC: Harbor-UCLA Medical Center
ICU: intensive care unit
IRB: Institutional Review Board
LAC: Los Angeles County
LAC-USC: Los Angeles County-University of Southern California
OVMC: Olive View Medical Center
SUPPORT: Study to Understand Prognoses and Preferences for Outcomes and Risks of Treatment
TLT: time-limited trial
UCLA: University of California Los Angeles
