# Supplementary material for: Time-Limited Trials Among Critically Ill Patients With Advanced Medical Illnesses to Reduce Nonbeneficial Intensive Care Unit Treatments: Protocol for a Multicenter Quality Improvement Study
Source: JMIR Res Protoc. 2019 Nov 25;8(11):e16301. doi: 10.2196/16301 (PMC6902129; doi:10.2196/16301)
Supplement: Multimedia Appendix 2 [file resprot_v8i11e16301_app2.pdf]

TIME LIMITED TRIAL PROTOCOL (Initial Meeting)

We designed this protocol as a guide to use during family meetings for patients at risk for receiving non-beneficial ICU treatments. Think of the steps as signposts—you might find that certain things do not apply to your patient and meeting.

Family Meeting Steps	Sample Phrases
**1. Introduce everyone and the agenda for meeting** ☐	• Let's start with introductions. My name is [A] and my role is [B].<br>• The purpose of this meeting is to talk about [C, D, E…].<br>• Is there anything that you would like to cover in addition?
**2. Explain what is happening with patient** ☐	• Tell me what you understand of [patient]'s condition and the medical care he/she has received in the ICU.<br>• From our standpoint, here are the most important pieces of information so far [summarize ICU course and key findings].
**3. Define acute care needs and prognosis** ☐	• The most important treatments that [patient] is receiving are [summarize].<br>• Based on the information we have so far, our hope is he/she improves with these treatments. However, I am concerned that he/she may not. I believe that the likelihood that he/she responds to treatment is [prognosis and most likely outcomes of ICU care]<br><br>***If prognosis is grim, explain why and offer opportunity for questions…***<br>• I understand that this news (or prognosis) is difficult to hear. The reason we believe that the prognosis is poor is [explanation].<br>• I would like to pause here and give you the opportunity to ask questions before we continue.
**4. Empathize with each person, dignify emotions**	• I can see that you are concerned about [A]<br>• We are impressed and grateful that you are here to support [patient].
**5. Highlight the patient's voice and elicit his/her values and preferences** ☐	• Given his/her current condition, if [patient] could speak, what do you think he/she would say about his/her situation?<br>• What would [patient] say about what he/she would like to avoid?<br>• In terms of quality of life, what are the most important things to him/her?<br>• Would [patient] be okay with the most likely outcome of this ICU care?<br>• Would [patient] be okay with undergoing these invasive treatments?<br><br>***If patient would forgo ICU treatments, recommend transition to comfort-focused care.***<br><br>***If patient would continue ICU treatments, continue to next step.***
**6. Plan a time limited trial together** ☐	• It sounds like [patient] would be okay with ICU treatments right now, but if it looked like they were not helping perhaps it would be different story.<br>• I would like to make a recommendation…

	• Let's see how [patient] responds to these ICU treatments. Although some of these treatments may be cause some discomfort, it would give us the best opportunity to see how [patient] responds to them.<br>• The following information should help us decide whether there is improvement or not [define markers of improvement/worsening].<br>• If [patient] improves we should continue aggressive care and see how much overall improvement there will be.<br>• However, if [patient]'s condition worsens, we need to consider the possibility that ICU treatments he/she is receiving may not be able to achieve our goals of care<br>• Our concern in such a situation would be that [patient] would be at risk for suffering through uncomfortable, invasive treatments without benefit.<br>• In such circumstances, most patients and family members chose to change the goals of the ICU care towards focusing on comfort, recognizing that invasive treatments are unlikely to reverse the illness.<br>• Of course, it is our hope that [patient] improves, but we mention these potential scenarios to emphasize that we should hope for the best, but also prepare for the worst.
**7. Allow reflection, questions and concerns**<br>☐	• I'd like to hear your [everyone's] thoughts about the plan
**8. Set a timeline to meet again**<br>☐	• Based on our plan, I would like to suggest that we meet again in [X] to discuss how [patient] is doing.<br>• If there are any urgent changes in his/her condition, we will notify you immediately.
**9. Conclude meeting**	• Thank you for taking the time to meet with us.<br>• It is encouraging for us to see that [patient] has your support.<br>• We look forward to speaking with you soon.

*Family Meeting format adapted from VitalTalk (vitaltalk.org)

TIME LIMITED TRIAL PROTOCOL (Follow-up Meeting)

We designed this protocol as a guide to use during **follow-up** family meetings for patients at risk for receiving non-beneficial ICU treatments. Think of the steps as signposts—you might find that certain things do not apply to your patient and meeting.

Family Meeting Steps	Sample Phrases
**1. Introduce everyone and the agenda for meeting** ☐	• Let's start with introductions. My name is [A] and my role is [B]. *(if there are new people who you have not met)*<br>• The purpose of this meeting is to follow-up on our previous meeting and discuss how [patient] has been doing.
**2. Re-cap last meeting and review new information** ☐	• Tell me what you recall from our last meeting.<br>• Since our last meeting, the most important news is that [summarize overall trajectory in one sentence].
**3. Update acute care needs and prognosis** ☐	• In our last meeting, we mentioned that we will be using certain information to decide whether [patient] is better or worse [remind surrogates of which tests/outcomes].<br>• Since that meeting, those results are [summarize].<br>• Based on the results of these tests, we believe that [patient] is doing [better, worse, same… explain clinical trajectory].<br>• With this additional information we now have, I believe that ICU treatments are [helping, not helping, harming]<br><br>***If prognosis is grim, explain why and offer opportunity for questions (below).***<br>• I understand that this news (or prognosis) is difficult to hear. The reason we believe that the prognosis is poor is [explanation].<br>• I would like to pause here and give you the opportunity to ask questions before we continue.
**4. Highlight the patient's voice and elicit his/her values and preferences** ☐	• As you know, we like to maintain the perspective of what [patient] would have wanted if he/she could make decisions for him/herself.<br>• Based on the information I have given you, do you think [patient] would be okay with the treatments he/she is receiving given the most likely outcome of this ICU care? Why or why not?<br><br>***If patient would forgo ICU treatments, recommend transition to comfort-focused care.***<br><br>***If patient would continue ICU treatments, continue to next step.***
**5. Plan another time limited trial together** ☐	• It sounds like [patient] would be okay with continuing ICU treatments for now.<br>• Let's see how [patient] responds to ICU treatments a bit longer.<br>• The following information should help us decide whether there is improvement or not [define markers of improvement/worsening].<br>• I hope we will see some improvement, and we would certainly enjoy the opportunity to celebrate this with you.

	• However, if [patient]'s condition continues to worsen, it will be important to understand what that means.<br>• From a medical perspective, it would mean that [patient] is very unlikely to recover. What I mean by this is that he/she may die from the underlying illnesses or be left in a state of health that may be against his/her wishes.<br>• In that situation, our recommendation would be to consider discontinuing invasive treatments that are not helping him/her and transition of goals of care on maintaining comfort.<br>• This recommendation would be a way to reduce suffering by focusing on things that affect his/her comfort and minimizing the use of invasive treatments that will not improve the outcome of his illness.
**6. Allow reflection, questions and concerns**<br>☐	• I'd like to hear everyone's thoughts about the plan
**7. Set a timeline to meet again**<br>☐	• Based on our plan, I would like to suggest that we meet again in [X] to discuss how [patient] is doing.<br>• If there are any urgent changes in his/her condition, we will notify you immediately.
**8. Conclude meeting**	• Thank you for taking the time to meet with us.<br>• We look forward to speaking with you soon.

*Family Meeting format adapted from VitalTalk (vitaltalk.org)
