# Supplementary material for: Time-Limited Trials Among Critically Ill Patients With Advanced Medical Illnesses to Reduce Nonbeneficial Intensive Care Unit Treatments: Protocol for a Multicenter Quality Improvement Study
Source: JMIR Res Protoc. 2019 Nov 25;8(11):e16301. doi: 10.2196/16301 (PMC6902129; doi:10.2196/16301)
Supplement: Multimedia Appendix 3 [file resprot_v8i11e16301_app3.pdf]

April 7, 2017

Re: Application for DHS-UCLA-USC Implementation Science Pilot Program

Dear Dr. Dong Chang,

Congratulations! We are pleased to inform you that your proposal, **"Time Limited Trials to Reduce Non-Beneficial Intensive Care Unit Treatments Among Critically-Ill Patients with Advanced Medical Illnesses"** submitted to the UCLA Clinical and Translational Science Institute, the Southern California Clinical and Translational Science Institute, and the Los Angeles County Department of Health Services (LAC DHS), has been selected for funding.

You will receive $75,000 in UCLA CTSI voucher funding which must be utilized exclusively for this project in accordance with the proposed budget, and will be available for the 12-month period beginning April 1, 2017. Please complete and return the attached CTSI Statement of Acceptance form to Jovi Ednave at jednave@mednet.ucla.edu. **Receipt of the form and compliance with its requirements must occur prior to our release of funds.**

Other award requirements are as follows:

- Any publications or documents that benefit from this award are required to acknowledge the funding source. We would suggest including the following language: "This project received support from the **NIH NCATS UCLA CTSI Grant Number UL1TR0001881.**"
- As a CTSI-funded researcher, you will be asked to participate in team-science workshop trainings. We will follow up with next steps to coordinate the team-science session.

Dr. Hal Yee is interested in discussing next steps for your project in person or via teleconference. We ask that you please contact Dr. Yee directly at hyee@dhs.lacounty.gov to arrange this meeting. The reviewer comments from your application are also enclosed for your reference.

Sincerely,

Hal Yee, Jr., M.D., Ph.D.
Chief Medical Officer, Los Angeles County Department of Health Services

Steven M. Dubinett, M.D.
Director, UCLA Clinical and Translational Science Institute

Thomas A. Buchanan, M.D.
Director, Southern California Clinical and Translational Science Institute

---

DHS-USC-UCLA Implementation Science Pilot Review Form

Instructions: After reviewing your assigned application, provide five scores in the tables below – one for each criteria, and one overall score. Use the same scale for all scores. Please use whole integers only. Comments for criteria scores are optional; comments for the overall score are required. Save and submit this completed form to Allison.orechwa@med.usc.edu. Your comments will remain completely confidential.

Due Date: March 6

Scoring Scale:

Score	Description
1	Outstanding
2	Excellent
3	Good
4	Fair
5	Poor

Criteria Scores:

How well does this project…	Score	Comments (optional)
1. Test a solution for a problem in healthcare (as opposed to measuring or understanding a problem or developing a solution)?	1	
2. Test solution(s) that is/are aligned with the DHS's specific infrastructure, business approach and operations, as opposed to a theoretically good idea?	1	
3. Involve broad participation across sites and disciplines, as opposed to a single specialty or single hospital or clinic?	1	
4. Focus on effectiveness (i.e., whether the intervention works in real-life) rather than efficacy (whether the intervention works under the controlled conditions of a study)?	1	

Overall Score:

Overall Score	Comments
1	This proposal builds upon the Harbor- and Reagan-UCLA based investigative team's previous published work on a DHS system critical issue of unnecessary use of higher level of care. Harbor PI has worked to incorporate other DHS hospitals (LAC-USC, OVMC) in the intervention arm, enabling generalizeabilty to DHS ICU settings. Study design has high likelihood of successfully developing and definitively evaluating a potential intervention in a real-world setting to address futile care in ICUs of all DHS hospitals.

DHS-USC-UCLA Implementation Science Pilot Review Form

Instructions: After reviewing your assigned application, provide five scores in the tables below – one for each criteria, and one overall score. Use the same scale for all scores. Please use whole integers only. Comments for criteria scores are optional; comments for the overall score are required. Save and submit this completed form to Allison.orechwa@med.usc.edu. Your comments will remain completely confidential.

Due Date: March 6

Scoring Scale:

Score	Description
1	Outstanding
2	Excellent
3	Good
4	Fair
5	Poor

Criteria Scores:

How well does this project…	Score	Comments (optional)
1. Test a solution for a problem in healthcare (as opposed to measuring or understanding a problem or developing a solution)?	1	Well defined problem, large impact. Also, the topic of communication is generally an important topic to a health system/DHS so there may be learning within this project that also applies to other DHS priorities, PRIME projects etc. (just as added value)
2. Test solution(s) that is/are aligned with the DHS's specific infrastructure, business approach and operations, as opposed to a theoretically good idea?	1	Has a strong design for learning within the system; also, engaging people within the system in designing/giving input to the solutions.
3. Involve broad participation across sites and disciplines, as opposed to a single specialty or single hospital or clinic?	1	The proposal shows that the team has given thought to scale within the institution and also spread to other institutions; has consulted and engaged those other institutions already
4. Focus on effectiveness (i.e., whether the intervention works in real-life) rather than efficacy (whether the intervention works under the controlled conditions of a study)?	1	Emphasis on change to existing practice; all testing and learning is done within the system, with DHS provider and improvement team collaboration

Overall Score:

Overall Score	Comments
1	This project appears ideally suited to come up with solutions that can work at scale and spread within DHS. I think that a time series format would not only help with the learning process but also would enable the investigators to track change in real time, and would also help with ensuring that gains that are made are held over time (also, presumably, of great interest to DHS). Using statistical process control would enable the team to interpret change over time without the power calculations that can slow learning. This might be a discussion point with the team, to enhance their proposal. Also, the team might build in an element of sharing initial results with a subgroup of clinicians/care team members rather than having input at the outset in a focus group and not coming back to that larger group until the changes are complete. Again this is just a suggestion for increasing the chances that the final protocol/solution will be feasible and designed to work for everyone who needs to use it.
